# Isolated Ventricular Noncompaction Cardiomyopathy Presenting as Recurrent Syncope

**DOI:** 10.1155/2016/3742171

**Published:** 2016-12-26

**Authors:** Sukhdeep Bhogal, Vatsal Ladia, Puja Sitwala, Kais AlBalbissi, Timir Paul

**Affiliations:** ^1^Department of Internal Medicine, East Tennessee State University, Johnson City, TN 37604, USA; ^2^Division of Cardiology, East Tennessee State University, Johnson City, TN 37604, USA

## Abstract

Isolated ventricular noncompaction (IVNC) occurs because of interruption of trabecular morphogenesis in the myocardium leading to ventricular noncompaction. Patients present with heart failure or with systemic complications secondary to thromboembolism or arrhythmias. High index of suspicion is necessary for early diagnosis. We present a case of 48-year-old male with unexplained recurrent syncope who was eventually diagnosed with IVNC.

## 1. Introduction

Cardiomyopathies have been classified based on morphological and functional phenotypes and each phenotype is further subdivided into genetic or nongenetic types. It is a rare disease that occurs due to the interruption of normal endomyocardial morphogenesis leading to noncompaction cardiomyopathy. It is almost always associated with other congenital anomalies like cyanotic heart disease or coronary artery anomalies. By contrast, isolated ventricular noncompaction (IVNC), is persistence embryonic isolated myocardial sinusoids in the nonexistence of other cardiac anomalies. Establishing correct diagnosis is pertinent for proper management and improving prognostic outcomes.

## 2. Case Presentation

**Figure 1:**
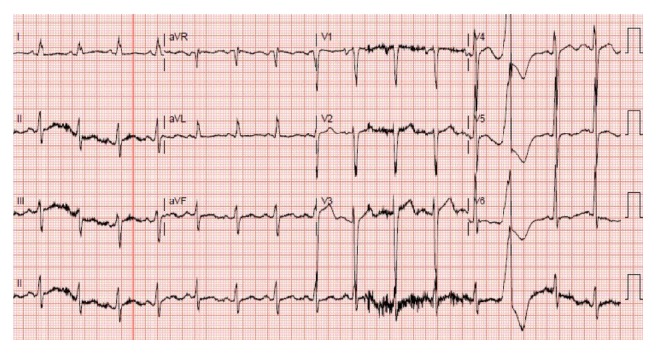
EKG showing sinus rhythm with premature ventricular complexes and nonspecific T wave changes.

A 48-year-old white male presented to our hospital with three-month history of shortness of breath and recurrent syncope episodes. He reported that his shortness of breath has been gradually progressive limiting his activity up to the level of walking short distances only. He denied history of hypertension or diabetes. Family history was significant for his sister in her fifties who died of heart disease. On examination, blood pressure was 118/87 mmHg, pulse was 96 beats/min, and respiratory rate was 18 breaths/min. On auscultation, there was normal S1, S2 with no gallops, or murmur noticed. Other physical examination was unremarkable. Chest X-ray was unremarkable as well. Electrocardiogram (EKG) showed sinus rhythm with premature ventricular complexes and nonspecific T wave changes (Figure 1). He reported recurrent admissions for the syncope in last 6 months. Cardiology was consulted for unexplained symptoms and given family history of premature heart disease.

**Figure 2:**
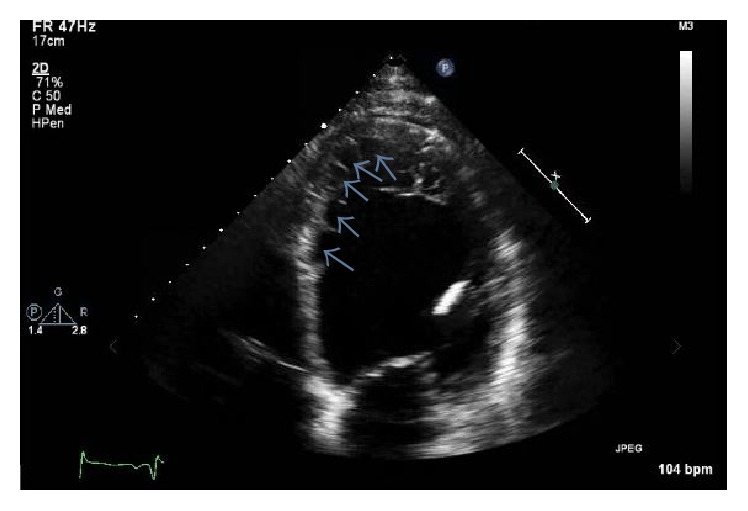
Two-dimensional echocardiogram in our patient demonstrating prominent trabeculations and deep intratrabecular recesses (marked by arrows).

**Figure 3:**
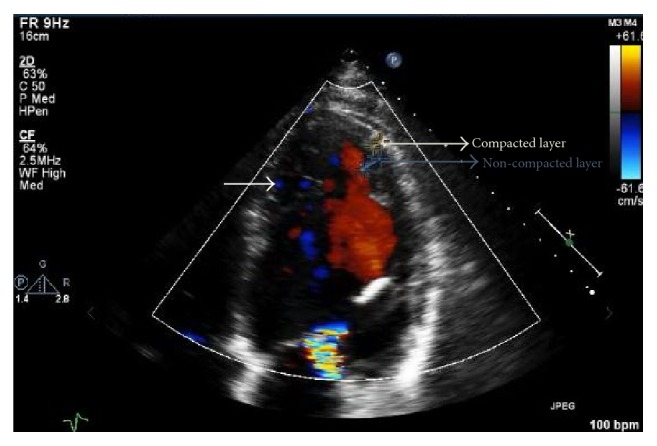
Color doppler echocardiogram demonstrating blood flow in the deep intertrabecular space (white arrow). Also, blue marking on the left ventricular wall showing noncompacted layer measuring 9.6 mm and yellow marking showing compacted layer measuring 4.4 mm with resulting ratio of noncompacted to compacted layer >2 at the end of systole.

**Figure 4:**
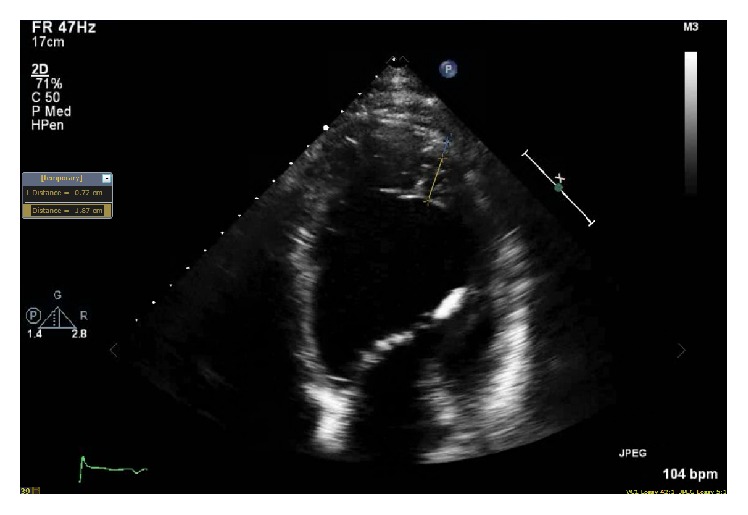
Apical four-chamber view of echocardiography demonstrating the end diastolic ratio of noncompacted layer 18.7 mm (yellow marking) and compacted layer 7.2 mm (blue marking) with resultant ratio of >2.

For further evaluation, echocardiogram was done that showed appearance of trabeculations and deep intertrabecular recesses (Figure 2), blood flow from ventricular chamber into intertrabecular spaces on doppler imaging (Figure 3), systolic thickness of compacted layer less than 8 mm (Figure 3), and ratio of noncompacted to compacted layer of >2 at end of systole (Figure 3) as well as diastole (Figure 4) consistent with noncompaction. There were no coexisting cardiac abnormalities seen. Also, left ventricular ejection fraction was evaluated below 20% along with severely increased left ventricular size and grade 1 diastolic dysfunction based on doppler filling pattern.

To further evaluate this cardiomyopathy, coronary catheterization was performed that revealed 50% stenosis of proximal left anterior descending, 50% stenosis of mid circumflex artery, and 50% stenosis of proximal right coronary artery which did not explain the aforementioned severely reduced cardiomyopathy. Patient was started on maximal medical management for treatment of his heart failure and was discharged on lifevest initially. He was then considered as a candidate of single chamber automated implantable cardioverter defibrillator (AICD) after repeating echocardiogram after 3 months with no improvement in ejection fraction. Following AICD implantation, he reported resolution of syncope on follow-up at 3- and 6-month interval.

## 3. Discussion

### 3.1. Embryology and Pathophysiology

During embryology, human heart is the first organ to start functioning even before structural development is complete [1]. Human heart undergoes various morphological events during its development. These events include heart tube formation, looping, growth, and development of heart chambers along with endocardium and septal morphogenesis. The first step is the migration of two primordial epithelial tubes, followed by fusion leading to formation of heart tube [2]. One of the theories suggests that epithelial cells organize themselves in the form of a cylindrical sheet resulting in formation of hollow heart tube [3]. The second step of loop formation signifies the complexity of heart development and possible mechanisms include the enlargement of cardiac jelly or dissimilar growth of heart tube [1]. Third step is the trabecular formation circumferentially along the heart tube in the form of ridge like outgrowths of endocardium followed by trabecular morphogenesis [1, 4]. This process of trabeculation is of paramount importance to uniformly distribute transmural stress and to increase the blood flow in the myocardial walls [3]. Fourth step includes the valvulogenesis from endocardial cushions. Subsequently, septation of heart is believed to occur as a result of intracardiac pressure gradient between two spiral blood streams during the process of development [5]. The process of compaction proceeds from epicardium to endocardium. The arrest of trabecular morphogenesis is believed to be underlying mechanism that leads to noncompaction of the myocardium.

### 3.2. Epidemiology and Genetics

Noncompaction cardiomyopathy is more common in men as compared to women. The real prevalence is still unknown, but based on echocardiogram reports, its prevalence is 0.014% [6]. Both familial and sporadic forms have been described. Familial is mainly reported as X linked disorder of inheritance. The genes associated with sporadic forms have not been identified yet. By contrast, familial forms are found to associate with the mutation of G4.5 gene of Xq28 chromosome segment [7]. This same segment is also linked to other myopathies including Barth syndrome and Emery-Dreifuss muscular dystrophy. Genetic studies in mice model have shown that deficiency of FKBP12 (a cytoplasmic protein) alters the process of trabeculation leading to hypertrabeculation and noncompaction [8]. Besides this, acquired noncompaction has also been reported in association with neurological disorders such as myotonic dystrophy [9]. Further research is warranted for better understanding of underlying molecular mechanism leading to noncompaction.

### 3.3. Clinical Features

Clinical features vary depending upon the severity of disease. The main clinical feature is heart failure and others include arrhythmias and thromboembolic events. Both systolic and diastolic heart failure can occur. In a cohort study of IVNC, systolic dysfunction is noticed in about 63% of cases [10]. Diastolic dysfunction is believed to be caused by restrictive filling and loss of relaxation mechanism of ventricle secondary to hypertrabeculation [6], while the mechanism for systolic dysfunction is unclear but is thought to be caused by alteration of blood supply in transmural and particularly in subendocardium causing ischemia secondary to numerous prominent trabeculae and their deep recesses [10]. Arrhythmias such as atrial fibrillation and ventricular tachyarrhythmias are common and cases of sudden cardiac death have been reported [10, 11]. In a study by Ichida et al. [12], abnormalities in electrocardiogram with ST depression and T wave inversion along with right bundle branch block are seen in 88% of cases. The same study reported the incidence of Wolff-Parkinson-White syndrome in 15% cases. Various thromboembolic complications (stroke, transient ischemic attacks, and pulmonary embolism) have been reported and are attributed to systolic dysfunction and atrial fibrillation [6]. In a systematic review of 241 patients, syncopal events were reported in 9% of cases [13]. Similarly, unexplained recurrent syncopal events lead to the diagnostic workup in our patient. Altogether, clinical manifestation of IVNC varies from asymptomatic presentation to heart failure, arrhythmias, syncopal events, and associated systemic complications such as thromboembolism and sudden cardiac death.

### 3.4. Diagnosis

Initially, various echocardiographic diagnostic criteria (Jenni et al. [14], Stöllberger) were proposed for diagnosis of IVNC. However, the analysis of these criteria by Kohli et al. in 2008 [15] revealed that they were too sensitive, particularly in black population leading to overdiagnosis of IVNC. For this reason, Stöllberger et al. [16] in 2012 refined the criteria and Gebhard et al. [17] added additional criterion to further improve the sensitivity of echocardiographic criteria. Currently, echocardiographic diagnosis [18] is based on combination of Jenni et al. (a–d), Stöllberger et al. (e), and Gebhard et al. (f), as seen in our patient:

- Appearance of prominent trabeculations and deep intertrabecular recesses principally in apical, mid-lateral, and mid-inferior region
- Direct blood flow from the ventricular chamber into intertrabecular spaces, seen on color Doppler imaging
- Absence of coexisting cardiac abnormalities
- The maximal end systolic ratio of noncompacted layer of myocardium to compacted layer of myocardium above 2
- The end diastolic ratio of noncompacted layer of myocardium to compacted layer of myocardium above 2 in apical four chamber view
- The fact that systolic thickness of compacted layer of myocardium less than 8 mm may aid the differentiation of IVNC from normal heart as well as hypertrophic one

The presence of above criteria makes the diagnosis highly probable, as gold standard is still lacking. Moreover, echocardiography poses certain pitfalls such as operator dependency and difficulty in visualizing the apex of myocardium [19].

Besides echocardiography, other diagnostic modality includes the use of magnetic resonance Imaging (MRI). Cardiac MRI is helpful in diagnosing subendocardial perfusion defects, assessing the size as well as localization of noncompaction [20]. Another advantage of cardiac MRI is proper view of apical region of ventricle which cannot be visualized accurately with echocardiogram, making it an important investigative tool to diagnose and confirmation noncompaction cardiomyopathy [21]. It can be particularly helpful in patients with inadequate echocardiographic imaging [22]. Positron emission tomography has been used in assessing the transmural perfusion defects attributed to noncompacted myocardium. Cardiac catheterization in some of these patients has demonstrated decreased ejection fraction or elevated pulmonary arterial hypertension.

### 3.5. Management

The treatment focuses on underlying symptoms and prevention of complications. Supportive care for both diastolic and systolic heart failure along with educating patient should be considered. Standardized treatments include the use of beta blockers, angiotensin converting enzyme inhibitors, and aspirin. Oral anticoagulation therapy is warranted in patients with history of atrial fibrillation and thromboembolism and those with ejection fraction of less than 40% [21]. Symptomatic patients with impaired ejection fraction should be refrained from participating in competitive sports. AICD, cardiac resynchronization therapy, and evaluation for heart transplant should be considered in patients with NYHA class III-IV heart failure and permanent atrial fibrillation or with bundle branch block [23]. Considering NYHA class III symptoms, low ejection fraction, and recurrent syncope, we proceeded with AICD implantation in our patient. Since the studies have shown familial association, screening echocardiography for the first-degree relatives was advised [24]. Refractory heart failure is managed with heart transplantation. Cases of successful heart transplantation have been reported to improve survival outcomes [25, 26].

## 4. Conclusion

Recurrent syncope in patients with IVNC is a relatively rare presentation. We propose that IVNC should be considered in the differential diagnosis of unexplained recurrent syncope particularly in young patients. Also, AICD implantation is a feasible option for patients with heart failure or low ejection fraction or maybe with recurrent syncope.
